# One-year survival of patients admitted for sepsis to intensive care units in Colombia

**DOI:** 10.1186/s12879-024-09584-7

**Published:** 2024-07-09

**Authors:** Henry Oliveros, Eduardo Tuta-Quintero, Mariana Piñeros, Alexander Guesguan, Luis F. Reyes

**Affiliations:** 1 School of Medicine, Universidad de La Sabana, Km 7, Autonorte de Bogota, Chía, Cundinamarca 250001 Colombia; 2 Centro Quirúrgico de La Sabana, Bogotá, Colombia

**Keywords:** Sepsis, Mortality, Critical care, Intensive care

## Abstract

**Background:**

Sepsis is a frequent cause of admission to intensive care units (ICUs). High mortality rates are estimated globally, and in our country, few studies have reported one-year survival. The objective of this study is to determine one-year survival in patients with sepsis admitted to the ICU in Colombia, compared with the survival of patients admitted for other conditions.

**Methods:**

Retrospective cohort study using administrative databases from the Ministry of Health of Colombia. One-year survival and the adjusted hazard ratio for survival, adjusted for comorbidities included in the Charlson Index, were determined using a Cox proportional hazards model for patients admitted for other causes as well as for those admitted for sepsis. This was then compared with an inverse propensity score weighting model.

**Results:**

A total of 116.407 patients were initially admitted to the ICUs, with 12.056 (10.36%) diagnosed with sepsis. Within the first year, 4.428 (36.73%) patients died due to sepsis. Age and male gender were associated with an increased risk of death from sepsis, and the covariates associated with one-year mortality were as follows: age over 80 years with HR 9.91 (95% CI: 9.22–10.65), renal disease with HR 3.16 (95% CI: 3.03–3.29), primary tumoral disease with HR 2.07 (95% CI: 1.92–2.23), liver disease with HR 2.27 (95% CI: 2.07–2.50), and metastatic solid tumor with HR 2.03 (95% CI: 1.92–2.15).

**Conclusion:**

This study revealed a high one-year sepsis mortality rate in the population, associated with variables such as age over 80 years, the presence of renal disease, liver disease, connective tissue diseases, and cancer. Men exhibited higher mortality compared to women.

**Supplementary Information:**

The online version contains supplementary material available at 10.1186/s12879-024-09584-7.

## Introduction

Sepsis continues to be one of the leading causes of patient admissions to intensive care units (ICUs) [1]. The estimated incidence of sepsis in the last decade is 276 cases per 100,000 person-years [2]. Hospital mortality rates range from 12.5 to 32.2%, which may vary depending on the sepsis origin, comorbidities, and geographical regions such as North America, Europe, and Oceania [3, 4]. Rudd et al. [5]. described 48.9 million incident cases and 11.0 million sepsis-related deaths in a global disease burden study with administrative data from 1990 to 2017. Despite efforts made for timely diagnosis and treatment, a reduction in sepsis incidence and mortality has not been achieved [6–9].

Abu-Kaf et al. [10], in 2018, reported a mortality rate of 4.5% for patients diagnosed with sepsis, compared to 0.7% for those admitted to the ICU for other reasons. Bauer et al. [11], in a systematic review of 170 studies, described sepsis mortality rates at 30 and 90 days ranging from 3.26 to 46.71% and 13.04–56.67%, respectively. Liu et al. [12] reported sepsis mortality in China between 20.6% and 50.8%.

Characterizing the burden of sepsis in different regions and identifying influencing factors could enable the development of strategies for timely diagnosis and treatment, positively impacting clinical outcomes [9, 13]. This study aims to determine the mortality of sepsis patients admitted to ICUs in Colombia and their one-year survival.

## Methods

A retrospective cohort study was conducted using administrative data from the Integrated Social Protection Information System (SISPRO) of the Colombian Ministry of Health.

### Population

Patients over 18 years of age who were admitted to approximately 300 ICUs across Colombian territory from January 1 to December 31, 2019, with a diagnosis of sepsis were included. In the case of patients with multiple admissions, data from the first hospitalization were used. Patients were identified by procedure codes related to ICU admission.

### Data sources

Data were sourced from the capitation unit (UPC) databases of the Integrated Protection System (SISPRO) of the Colombian Ministry of Health. This database comprises reports of services provided by health promotion companies to 22.4 million Colombians, representing 45% of the population enrolled in the contributory regime. The database is highly standardized and includes service codes (CUPS), service date, age, gender, insurance provider, municipality, and ICD-10 codes (See supplementary Table 1). Mortality information, date of death, and diagnoses related to the cause of death were obtained from death certificates (RUAF). The datasets utilized and/or analyzed in the current study are available upon reasonable request from the corresponding author.

### Studied variables

The primary outcome was one-year mortality and the diagnosis of sepsis. Other variables considered included demographic characteristics such as age, gender, and place of origin. Coexisting conditions identified using the Charlson Index, validated for the Colombian population [14], were also included. The mortality records were efficiently integrated with hospitalization records and death dates by using a unique identification number and death certificates (RUAF) to determine one-year mortality. All patients were followed for a minimum of one year, with administrative censoring as of December 31, 2020, or until the occurrence of the death event.

### Statistical analysis

Baseline demographic and clinical characteristics for patients with and without sepsis were determined and compared using standardized differences to assess balance between the two groups. Hazard ratios (HR) for one-year survival were then calculated between patients with sepsis and those without sepsis. To control for confounding due to systematic differences between patients with and without sepsis with respect to survival, demographic characteristics, and coexisting conditions were included in the Cox proportional hazards model. These variables included age, gender, hypertension, diabetes mellitus, cerebrovascular disease, connective tissue disease, liver disease, cancer, neoplastic disease, and metastatic disease.

Finally, to assess the consistency of the Cox model, it was compared with hazard coefficients obtained from a pseudo-population created by weighting inverse scores from propensity scores derived from a logistic regression model of the probability of sepsis occurrence based on patient characteristics and comorbidities. Data processing and statistical analyses were performed using the following software: EmEditor 22.3, Python 3.9, and the R statistical package (See supplementary Table 2).

## Results

**Fig. 1:**
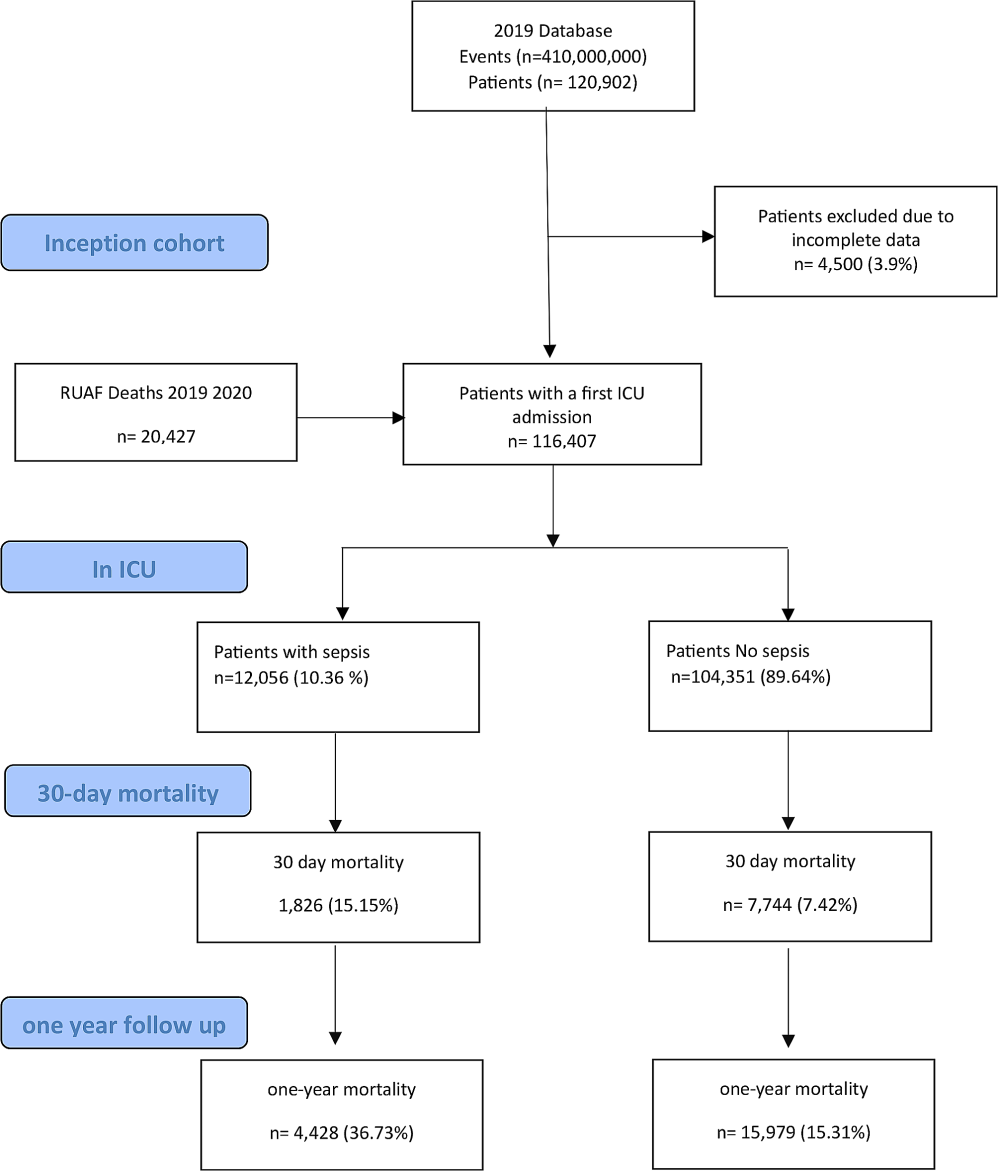
Flowchart of included subjects based on administrative health data. *Notes* ICU: Intensive care unit

Out of a total of 120.902 patients admitted for the first time to 300 ICUs in Colombian territory during the period from January 1 to December 31, 2019. 4.500 patients were excluded due to incomplete data, leaving a total of 116.407 patients. We identified 12.056 (10.36%) patients with a sepsis diagnosis, which corresponds to 54.8 cases per 100.000 person-years. Among these, 1.826 (15.15%) patients died within 30 days of ICU admission, and 4.428 (36.73%) patients within the first year. Another 104.351 (89.64%) patients were admitted for other reasons, with 7.744 (7.42%) deaths at 30 days and 15.979 (17.31%) deaths within the year (Fig. 1).

### General population characteristics

The most prevalent comorbidities among the population of patients admitted to the ICU were arterial hypertension in 74.280 patients (63.79%), diabetes mellitus in 26.470 (22.73%), chronic obstructive pulmonary disease in 20.535 (17.63%), and acute myocardial infarction in 19.792 (17.01%). General population characteristics are presented in Table 1.

**Table 1:** General characteristics of patients hospitalized in the ICU in 2019

Baseline characteristics	Full sample *n*: 116,407	No sepsis *n* = 104,351	With sepsis *n* = 12,056	Standardized differences %
Age. mean (SD)	61.62 (17.48)	61.26 (17.3)	64.78 (18.73)	0.195
18–39 years s n (%)	15,122 (14.29)	13,560 (14.2)	1545 (13.9)	-0.009
40–49 years n (%)	8,883 (8.34)	8,166 (8.6)	711 (6.4)	-0.082
50–59 years n (%)	16,984 (15.95)	15,688 (16.5)	1292 (11.6)	-0.139
60–69 years n (%)	26,160 (23.63)	23,024 (24.2)	2,130 (19.2)	-0.121
70–79 years n (%)	22,895 (21.50)	20,380 (21.4)	2,512 (22.6)	0.030
> 80 years n (%)	17,440 (16.38)	14,519 (15.2)	2,915 (26.2)	0.274
Male gender. n (%)	57,497 (49.37)	51,669 (49.5)	5,801 (48.1)	-0.02
Myocardial infarct n (%)	19,792 (17.00)	17,932 (17.2)	1859 (15.4)	-0.048
Hypertension n (%)	74,280 (63.79)	67,165 (64.4)	7,113 (59.0)	-0.113
Congestive heart failure n (%)	3,599 (3.06)	3,097 (3.0)	462 (3.8)	0.042
Peripheral vascular disease n (%)	3,474 (2.98)	2,851 (2.7)	623 (5.2)	0.126
Cerebrovascular disease n (%)	8,478 (7.29)	7,225 (6.9)	1,253 (10.4)	0.123
Dementia n (%)	3,910 (3.36)	3,201 (3.1)	709 (5.9)	0.138
Chronic pulmonary disease n (%)	20,535 (17.63)	17,279 (16.6)	3,256 (27.0)	0.254
Connective tissue disease n (%)	5,410 (4.65)	4,818 (4.6)	592 (4.9)	0.007
Moderate or severe liver disease n (%)	1,063 (0.91)	817 (0.8)	246 (2.0)	0.106
Moderate or severe renal disease n (%)	6,230 (5,35)	4,723 (4,9)	865 (12.7)	0.288
Hemiplegia n (%)	215 (0.18)	164 (0.2)	51 (0.4)	0.051
primary tumoral disease* n (%)	1,770 (1.52)	1,378 (1.3)	392 (3.3)	0.127
Metastatic solid tumor n (%)	4,038 (3.47)	3,389 (3.2)	648 (5.4)	0.104
HIV/AIDS n (%)	1,883 (1.62)	1,547 (1.5)	336 (2.8)	0.082
Diabetes mellitus n (%)	26,470 (22.73)	22,982 (22.0)	3,486 (28.9)	0.158
30 - day mortality n (%)	9,588 (8.23)	7,744 (7.42)	1,826 (15.15)	-
1- year mortality n (%)	20,427 (17.54)	15,979 (15,31)	4,428 (36.73)	-

*Note* SD: standard deviation; HIV/SIDA: Human immunodeficiency virus/ acquired immunodeficiency syndrome

*Including leukemias and lymphomas

### One-year survival in sepsis patients

The crude one-year survival of sepsis patients had an HR of 2.71 (95% CI: 2.62–2.80). The adjusted HR was 1.81 (95% CI: 1.75–1.88), and the HR adjusted by IPW was 2.10 (95% CI: 2.02–2.18).

### Factors associated with one-year survival in ICU admitted patients

In the general population of patients admitted to the ICU, conditions associated with a higher risk of one-year mortality included renal disease (HR 3.17, 95% CI: 3.04–3.31), liver disease (HR 2.22, 95% CI: 2.00–2.46), primary tumoral disease (HR 2.07, 95% CI: 1.97–2.13), and metastatic solid tumor (HR 2.01, 95% CI: 1.90–2.13) (Table 2 and Supplementary Fig. 1).

**Table 2:** Hazard ratios for the analysis of one-year survival of patients admitted to the ICU, using an adjusted COX model

Independent variable	Hazard ratio	Confidence interval
Male	1.11	1.08–1.14
Age	1.05	1.05–1.05
Sepsis	1.81	1.75–1.88
Cerebrovascular disease	1.46	1.39–1.52
Congestive heart failure	1.27	1.19–1.36
Previous renal disease	3.17	3.04–3.31
Myocardial infarct	0.85	0.82–0.88
Peripheral vascular disease	1.04	0.97–1.11
Dementia	1.10	1.04–1.17
Chronic obstructive pulmonary disease	1.10	1.06–1.13
Connective tissue diseases	1.20	1.13–1.28
Liver disease	2.22	2.00–2.46
Hemiplegia	1.21	0.89–1.65
primary tumoral disease*	2.07	1.90–2.24
Metastatic solid tumor	2.01	1.90–2.13
HIV/AIDS	1.50	1.35–1.67
Mellitus diabetes	0.96	0.93–0.99

*Note* HIV/SIDA: Human immunodeficiency virus/ acquired immunodeficiency syndrome

*Including leukemias and lymphomas

### Factors associated with one-year survival in sepsis patients

In the population of sepsis patients, variables associated with one-year mortality were age over 80 years (HR 9.91, 95% CI: 9.22–10.65), renal disease (HR 3.16, 95% CI: 3.03–3.29), primary tumoral disease (HR 2.07, 95% CI: 1.92–2.23), liver disease (HR 2.27, 95% CI: 2.07–2.50), metastatic solid tumor (HR 2.03, 95% CI: 1.92–2.15), and connective tissue disease (HR 1.2, 95% CI: 1.16–1.32) (Table 3 and Supplementary Fig. 2).

**Table 3:** Hazard ratios via a Cox Regression Model in patients with and without sepsis

	Sepsis		No sepsis	
Basic features	Hazard ratio	95% confidence interval.	Hazard ratio	95% confidence interval.
40–49 years n (%)	1.40	1.28–1.55	1.35	1.22–1.50
50–59 years n (%)	1.78	1.64–1.94	1.70	1.56–1.86
60–69 years n (%)	2.79	2.59–3.00	2.70	2.50–2.93
70–79 years n (%)	4.79	4.45–5.14	4.90	4.53–5.30
> 80 years n (%)	9.91	9.22–10.65	10.4	9.69–11.35
Male	1.14	1.10–1.17	1.13	1.10–1.17
Cerebrovascular disease	1.48	1.42–1.55	1.61	1.53–1.68
Congestive heart failure	1.27	1.18–1.36	1.36	1.26–1.46
Previous renal disease	3.16	3.03–3.29	3.56	3.40–3.73
Myocardial infarct	0.85	0.82–0.89	0.87	0.84–0.91
Hypertension	0.50	0.48–0.52	0.48	0.46–0.50
Peripheral vascular disease	1.04	0.97–1.11	1.09	1.01–1.18
Dementia	1.08	1.01–1.14	1.10	1.03–1.17
Chronic obstructive pulmonary disease	1.07	1.04–1.11	1.12	1.08–1.16
Connective tissue diseases	1.24	1.16–1.32	1.20	1.12–1.29
Liver disease	2.28	2.07–2.50	2.67	2.40–2.97
Hemiplegia	1.14	0.87–1.48	1.57	1.17–2.13
primary tumoral disease*	2.07	1.92–2.23	2.34	2.16–2.54
Metastatic solid tumor	2.03	1.92–2.15	2.18	2.05–2.32
HIV/AIDS	1.42	1.28–1.57	1.44	1.38–1.62
Mellitus diabetes	0.96	0.93–0.99	1.01	0.97–1.05

*Note* HIV/SIDA: Human immunodeficiency virus/ acquired immunodeficiency syndrome

*Including leukemias and lymphomas

### Factors associated with one-year survival in no sepsis patients

In the population of sepsis patients, variables associated with one-year mortality were age over 80 years (HR 10.4, 95% CI: 9.69–11.35), renal disease (HR 3.56, 95% CI: 3.40–3.73), liver disease (HR 2.67, 95% CI: 2.40–2.97), primary tumoral disease (HR 2.34, 95% CI: 2.16–2.54), metastatic solid tumor (HR 2.18, 95% CI: 2.05–2.32), and connective tissue disease (HR 1.20, 95% CI: 1.12–1.29) (Table 3 and Supplementary Fig. 3).

### Propensity score matching and inverse weighting model

**Fig. 2:**
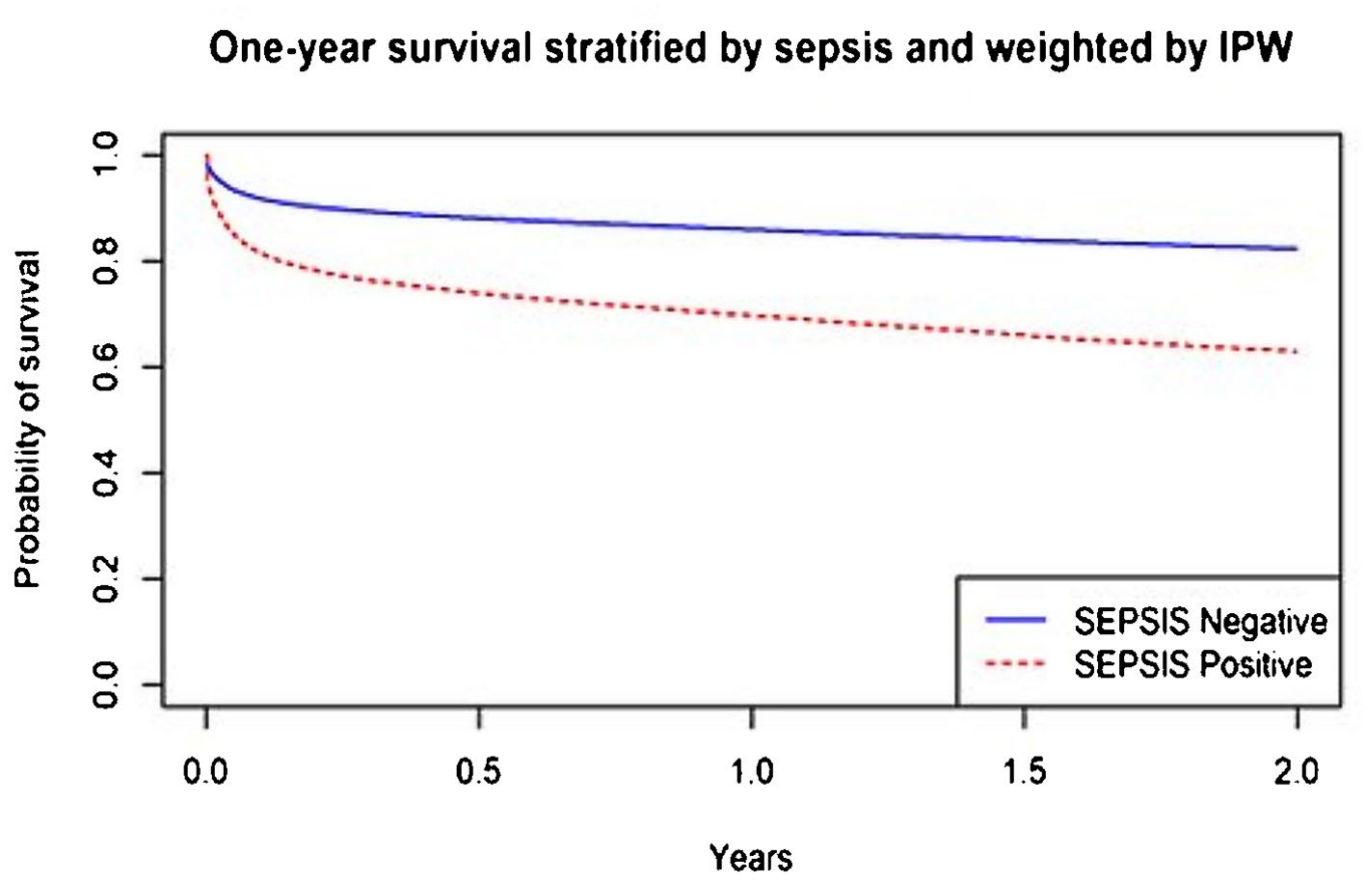
Survival of patients diagnosed with sepsis and without sepsis at one year. *Notes* IPW: inverse probability weight

After matching with propensity scores and calculating the inverse weight model (IPW), it was found that sepsis led to an increased risk of one-year mortality with an HR of 2.10 (95% CI: 2.02–2.18) (Table 4). Figure 2 displays one-year survival curves adjusted for Inverse Probability Weighting (IPW).

**Table 4:** Risk of mortality in patients with sepsis

Outcome	Crude HR (IC 95%)	Adjusted HR (IC 95%) *	IPW HR (IC 95%)
Mortality	2.71 (2.62–2.80)	1.81 (1.75–1.88)	2.10 (2.02–2.18)

*Note* HR: Hazar ratio; IPW: inverse probability weight

*Adjusted for age, sex, and Charlson comorbidity index variables

### One-year survival rate and regional mortality

The one-year survival rate in different regions of the country is described in the supplementary Fig. 4. It can be observed that males had an increased mortality risk, expressed with an adjusted HR of 1.11 (95% CI: 1.08–1.14), and age was up to 10 times higher for sepsis patients over 80 years.

## Discussion

The proportion of patients diagnosed with sepsis admitted to the ICU was 10.36% (12.054), representing a rate of 54.8 cases per 100.000 person-years in 2019. Both the proportion and the found rate align with what has been reported in the literature. The 30-day sepsis mortality of 15.2% falls within the wide range of mortality reported in various studies, which varies depending on coexisting conditions and the source of sepsis. Regarding comorbidities associated with one-year mortality in the entire patient cohort, renal disease was the leading factor, followed by cancer, liver disease, metastatic neoplastic disease, and, finally, connective tissue diseases. However, the variable with the highest hazard ratio for mortality was age over 80 years, with a ninefold higher risk for octogenarian patients compared to younger patients [13, 15–18].

Population-level estimates of sepsis incidence and mortality have commonly relied on administrative databases [7, 8]. Oh et al. [9] used data from the Korean National Health Insurance Service to examine patients hospitalized with a sepsis diagnosis. In 2007, the incidence rate was 173.8 cases per 100.000, which increased to 233.6 cases per 100-000 by 2016. Our study showed a rate of ICU-hospitalized patients of 54.8 cases per 100.000 person-years, similar to the rate reported by Fleischmann et al. in a meta-analysis encompassing 30 studies published from 1979 to 2019, which found a rate of 58 cases per 100.000 person-years.

Fleischmann et al. [13] described a lower annualized sepsis incidence between 2010 and 2015 in Germany compared to other studies. This difference was attributed to limitations and biases in administrative data, including omitted clinical diagnoses of sepsis, varying thresholds, and variability in disease diagnosis and coding practices. In our results, we used administrative data, which could have influenced the extrapolation of the findings to the general population, as described by Fleischmann. However, the algorithms used to identify sepsis patients considered not only ICD-10 codes but also the administration of broad-spectrum antibiotics, increasing sensitivity in patient identification.

Abe et al. [17] found that 67.0% of patients had at least one comorbidity, with the most common being diabetes mellitus (23.0%), malignancy (17.7%), cerebrovascular diseases (11.8%), and heart failure (10.8%). Our study aligns with diabetes being the most common coexisting disease in ICU-admitted patients, with chronic obstructive pulmonary disease and systemic arterial hypertension having the highest prevalence. Similar to overall ICU patient mortality, in patients with a sepsis diagnosis, age over 80 years was the variable with the highest one-year mortality risk. Strandberg et al. [19] found an association between age over 80 years and sepsis as the reason for ICU admission (HR = 3.87; 95% CI: 3.44–4.35) and the need for renal replacement therapy in the ICU (HR = 1.11, 95% CI: 1.01–1.21).

In our study, we observed higher mortality among male patients diagnosed with sepsis, while Sakr et al. [20] reported higher mortality in women with severe sepsis compared to men (63.5% vs. 46.4%, *p* = 0.007). Additionally, we found lower sepsis mortality in regions with more advanced healthcare infrastructure and intensive care facilities. These findings suggest that access to specialized medical care and adequate resources can positively influence sepsis patient outcomes.

Limitations of the study include the inability to determine sepsis severity upon ICU admission due to data source constraints. However, coexisting conditions reported in the Charlson Index were taken into account. Systematic differences between patients admitted with a sepsis diagnosis and those with other diagnoses were assessed through standardized mean differences, and inverse propensity score weighting was employed to adjust hazard ratios obtained.

## Conclusions

This study revealed a high one-year sepsis mortality rate in the population, associated with variables such as age over 80 years, the presence of renal disease, liver disease, connective tissue diseases, and cancer. Men exhibited higher mortality compared to women.

### Electronic supplementary material

Below is the link to the electronic supplementary material.

Supplementary Material 1

## Data Availability

The datasets used and/or analyzed during the current study are available from the corresponding author upon reasonable request.
