# Supplementary material for: One-year survival of patients admitted for sepsis to intensive care units in Colombia
Source: BMC Infect Dis. 2024 Jul 9;24:678. doi: 10.1186/s12879-024-09584-7 (PMC11232145; doi:10.1186/s12879-024-09584-7)
Supplement: Supplementary file 1 — Supplementary Material 1 [file 12879_2024_9584_MOESM1_ESM.docx]

**Supplementary figure 1.** Hazard survival ratio at one-year survival of patients admitted to the ICU.

**Supplementary figure 2.** Hazard survival ratio at one-year survival of patients with sepsis.

**Supplementary figure 3.** Hazard survival ratio at one-year survival of patients without sepsis.

**Supplementary figure 4.** Crude mortality map by regions in Colombia

| 1. Mortality at 30 days in patients admitted to the Intensive Care Unit   **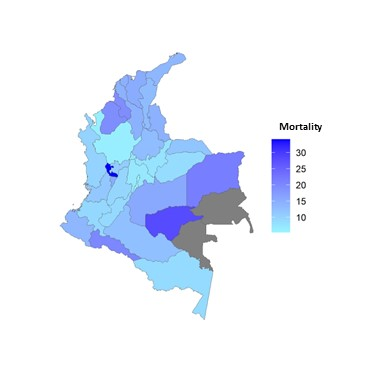** | 1. Mortality at 30 days in patients with sepsis   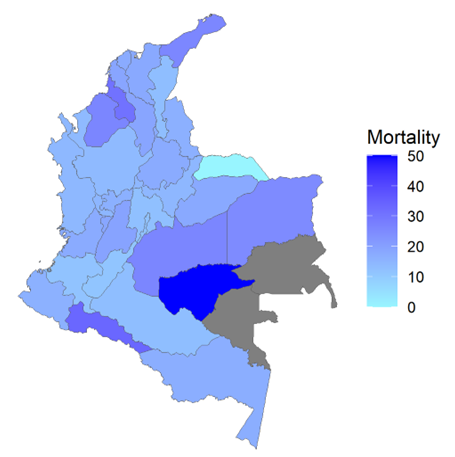 |
| --- | --- |

The color gradient illustrates the overall mortality by regions in Colombia, both for patients admitted to the Intensive Care Unit (A) and those who had sepsis (B). A lower frequency of mortality is evident in the central region of the country. In contrast, an increase in crude mortality is observed in the northeast and east of the country.

**Supplementary table 1.** Dimensions taken into account in the construction of the algorithms

| COMORBIDITY | ICD-10 CODES | CUPS AND MEDICATION |
| --- | --- | --- |
| MYOCARDIAL INFARCTION | I21* I22* I252 I255 | 360100 360101 360102 360102 360200 360201 360202 |
| CONGESTIVE HEART FAILURE | I110 I130 I132 I50* I420 I425 I429 I43* |  |
| PERIPHERAL VASCULAR DISEASE | K551 K558 K559 Z958 Z959 I70* I71* I720 I728 I739 I719 I731 I738 I771 I790 I792 | 380300 380910 383903 392204 392400 392501 392502 392503 392601 392602 392603 392604 392605 380300 392620 |
| STROKE | G45* G46* I60* I69* I67* I681 I682 I688 I694 I698 | 395010 380101 380110 380210 |
| DEMENTIA | F00* F01* F02* FO3* G30* G311 F03X F051 F010 | *RIVASTIGMINE* *DONEPEZIL* *GALANTAMINE* *MEMANTINE* *DONEPEZIL MEMANTINE* *BUPROPION* *AMANTADINE* |
| CHRONIC OBSTRUCTIVE PULMONARY DISEASE | J441 I278 I279 J4* J60* J61* J62* J63* J64* J66* J67* J684 J701 J703 J448 J449 J410 J411 J42X J431 J432 J438 J439 | *OXYGEN* |
| CONNECTIVE TISSUE DISEASE | M053 M058 M059 M060 M061 M063 M069 M050 M052 M051 M353 M320 M321 M328 M329 M353 M330 M331 M332 M339 M340 M341 M342 M348 M349 M353 | *METOTREXATE* *SULFASALAZINE* *LEFLUNOMIDE* *PENICILAMINE* *RITUXIMAB* *ABATACEPT* *ETANERCEPT* *INFLIXIMAB* *ADALIMUMAB* *CERTOLIZUMAB* *GOLIMUMAB* *TOCILIZUMAB* *TOFACIAXIN* *ANAKINRA* *TACROLIMUS* *ACTEMRA* |
| PEPTIC ULCER DISEASE | K270 K271 K272 K273 K274 K275 K276 K279 K250 K251 K252 K253 K254 K255 K256 K257 K259 K260 K261 K262 K263 K264 K265 K266 K267 K269 | S22224 441100 451301 451600 893904 901220 906022 906023 906024 438100 440100 |
| MILD LIVER DISEASE | K709 K702 K703 K717 K740 K742 K746 K740 K742 K746 K743 K744 K745 K730 K731 K738 K739 k70* k73* |  |
| TYPE 2 DIABETES | E100 E101 E106 E108 E109 E110 E111 E116 E118 E119 E120 E121 E126 E128 E29 E130 E131 E136 E138 E139 E140 E141 E146 E148 E149 E140 | *REPAGLINIDE* *NATEGLINIDE* *LIRAGLUTIDE* *EXENATIDE* *LIXIZENATIDE* *DULAGLUTIDE* *ACARBOSE* *MIGLITOL* *CLORPROPAMIDE* *TOLBUTAMIDE* *GLIBENCLAMIDE* *GLIMEPIRIDE* *GLICLAZIDE* *GLIBENS* *GLIPIZIDE* *INSULIN* *INSUMAN* *INSULEX* *INSULIN ASPART* *INSULIN GLULISINE* *INSULIN LISPRO* *INSULIN DETEMIR* *INSULIN GLARGINE* *INSULIN DEGLUDEC* *INSULIN NPH* *METFORMIN* *GEMIGLIPTIN* *EMPAGLIFLOZIN* *PHENFORMIN* *SITAGLIPTIN* *VILDAGLIPTIN* *LINAGLIPTIN* *ALOGLIPTIN* |
| PARAPLEGIA - HEMIPLEGIA | G81 G041 G820 G821 G822 |  |
| CHRONIC KIDNEY DISEASE | I120 I131 N03* N05* Z49* N18 N19 N25 N01 N074 N073 N072  N52 N19 N250 Z940 Z992 | 389500 394300 399501 549002 392701 392702 394200 549001 549012 549800 549801 549802 S22220 S22223 549800 |
| DIABETES MELLITUS WITH COMPLICATIONS | E10* E11* E12* E13* E14* H360 H280 G590 G632 M142 |  |
| ANY TUMOR INCLUDING LEUKEMIA/LYMPHOMA (WITHOUT MALIGNANT SKIN NEOPLASIA) | C000 C001 C002 C003 C004 C005 C006 C007 C008 C009 C01X C020 C021 C022 C023 C024 C029 C030 C031 C039 C040 C041 C049 C050 C051 C052 C059 C060 C061 C062 C069 C07X C080 C081 C089 C090 C091 C099 C101 C102 C103 C104 C883 C887 C889 C900 C901 C91* C92* C93* C94* C95* C96* |  |
| SEVERE LIVER DISEASE | K729 K766 K703 K767 K721 |  |
| METASTATIC SOLID TUMOUR | C780 C7* C8* C781 C782 C783 C784 C785 C786 C787 C788 C79 C790 C791 C792 C793 C794 C795 C796 C797 C798 |  |
| HIV | Z114 Z21X B200 B201 B202 B203 B204 B205 B206 B207 B208 B209 B210 B211 B212 B217 B219 B220 F028 R75X B220 B221 B24* | *ZIDOVUDINE* *LAMIVUDINE* *TENOFOVIR* *EMTRICITABIN* *DIDANOSINE* *NEVIRAPINE* *EFAVIRENZ* *ETRAVIRINE* *SAQUINAVIR* *LOPINAVIR* *ATAZANAVIR* *INDINAVIR* *NELFINAVIR* *RALTEGRAVIR* *ABACAVIR* *ABAMUNE* *RITONAVIR* *ATAZANAVIR* |
| SEPSIS | A418 A41 B377 A021 A403 A410 A414 A415 A400 A402 A408 A409 A418 A419 A542 B206 B951 B960 J158 J159 O85X T793 T814 T826 T847 T875 B371 B378 B379 | MEROPENEM *IMIPENEM* *VANCOMICINA* *TIGICICLINA TAZOBACTAM* *COLISTINA* *POLIMIXINA* *CEPEMIME* *DATOMICINA* *ERTAPENEM* |

# **Supplementary table 2.** R code used in the study

---

title: "SEPSIS"

author: "Henry Oliveros"

date: "2024-01-18"

output: html_document

---

**library**(survival) *# install.packages("survival")*

## Warning: package 'survival' was built under R version 4.2.3

install.packages("WeightIt")

## Installing package into 'C:/Users/henry/AppData/Local/R/win-library/4.2'

## (as 'lib' is unspecified)

## Warning: unable to access index for repository http://cran.r-project.org/src/contrib:

## download from 'http://cran.r-project.org/src/contrib/PACKAGES' failed

## Warning: package 'WeightIt' is not available for this version of R

##

## A version of this package for your version of R might be available elsewhere,

## see the ideas at

## https://cran.r-project.org/doc/manuals/r-patched/R-admin.html#Installing-packages

**library**(WeightIt)

## Warning: package 'WeightIt' was built under R version 4.2.3

**library**(dplyr)

## Warning: package 'dplyr' was built under R version 4.2.3

##

## Attaching package: 'dplyr'

## The following objects are masked from 'package:stats':

##

## filter, lag

## The following objects are masked from 'package:base':

##

## intersect, setdiff, setequal, union

**library**(ggplot2)

## Warning: package 'ggplot2' was built under R version 4.2.3

setwd("C:/Users/henry/Dropbox/SEPSIS")

long <- read.csv("sepsis.csv")

View(long)

**We check how many individuals have the outcome and how many are censored**

last <- long %>% group_by(personabasicaid) %>% filter(row_number() == n())

table(last$mortality, last$SEPSIS)

##

## 0 1

## 0 88372 7628

## 1 15979 4428

**Check how many start treatment**

inita <- long %>% filter(muerto_30 == 0) %>% group_by(personabasicaid) %>% filter(row_number() == n())

table(inita$SEPSIS)

##

## 0 1

## 96607 10230

**Preparation of Table No 1**

*# List of variables that will be included in table No. 1*

**library**(tableone)

## Warning: package 'tableone' was built under R version 4.2.2

myVars <- c("SEPSIS" , "ACV" , "ICC", "IR", "DEME", "EPOC", "CA", "HTA", "DM", "TC","EVP" ,"IAM","sex","age" , "EH" , "TM" , "HIV" , "PLEJIA" , "edad_c" , "year" , "mortality" )

***# List of categorical variables***

catVars <- c("SEPSIS" , "ACV" , "ICC", "IR", "DEME", "EPOC",

"CA", "HTA", "DM", "TC","EVP" ,"IAM","sexo", "EH" , "TM" , "VIH" , "PLEJIA" , "edad_c" , "mortality" )

***# List of continuous variables that are expressed as average and interquartile ranges***

medVars <- c("year")

edad_c <- factor("edad_c") ***# convert age as a factor***

***# Create table 1***

tab1 <- CreateTableOne(vars = myVars, ***# set descriptive variables***

strata = "SEPSIS", ***# Define the stratification variable*** data = long, ***# Database***

factorVars = catVars) ***# defines the categorical variables***

***# Print Table No 1***

print(tab1,

nonnormal = medVars,

formatOptions = list(big.mark = ","),

test = FALSE)

## Stratified by SEPSIS

## 0 1

## n 104,351 12,056

## SEPSIS = 1 (%) 0 ( 0.0) 12056 (100.0)

## ACV = 1 (%) 7225 ( 6.9) 1253 ( 10.4)

## ICC = 1 (%) 3097 ( 3.0) 462 ( 3.8)

## IR = 1 (%) 4723 ( 4.5) 1506 ( 12.5)

## DEME = 1 (%) 3201 ( 3.1) 709 ( 5.9)

## EPOC = 1 (%) 17279 (16.6) 3256 ( 27.0)

## CA = 1 (%) 1378 ( 1.3) 392 ( 3.3)

## HTA = 1 (%) 67165 (64.4) 7113 ( 59.0)

## DM = 1 (%) 22982 (22.0) 3486 ( 28.9)

## TC = 1 (%) 4818 ( 4.6) 592 ( 4.9)

## EVP = 1 (%) 2851 ( 2.7) 623 ( 5.2)

## IAM = 1 (%) 17932 (17.2) 1859 ( 15.4)

## sexo = 1 (%) 51669 (49.5) 5801 ( 48.1)

## edad (mean (SD)) 61.26 (17.30) 64.78 (18.73)

## EH = 1 (%) 817 ( 0.8) 246 ( 2.0)

## TM = 1 (%) 3389 ( 3.2) 648 ( 5.4)

## VIH = 1 (%) 1547 ( 1.5) 336 ( 2.8)

## PLEJIA = 1 (%) 164 ( 0.2) 51 ( 0.4)

## edad_c (%)

## 0 13560 (14.2) 1545 ( 13.9)

## 1 8166 ( 8.6) 711 ( 6.4)

## 2 15688 (16.5) 1292 ( 11.6)

## 3 23024 (24.2) 2130 ( 19.2)

## 4 20380 (21.4) 2512 ( 22.6)

## 5 14519 (15.2) 2915 ( 26.2)

## year (median [IQR]) 1.60 [1.24, 1.85] 1.37 [0.34, 1.70]

## mortality = 1 (%) 15979 (15.3) 4428 ( 36.7)

**Run a Cox proportional hazards model to calculate the crude hazard ratio between SEPSIS and DEATH AT ONE YEAR**

crudehr <- coxph(Surv(long$year, long$mortality) ~ SEPSIS, data = long, method = "efron", robust = TRUE, id = personabasicaid)

summary(crudehr)

## Call:

## coxph(formula = Surv(long$year, long$mortality) ~ SEPSIS, data = long,

## robust = TRUE, method = "efron", id = personabasicaid)

##

## n= 116407, number of events= 20407

## (45 observations deleted due to missingness)

##

## coef exp(coef) se(coef) robust se z Pr(>|z|)

## SEPSIS 0.99926 2.71626 0.01700 0.01677 59.6 <2e-16 ***

## ---

## Signif. codes: 0 '***' 0.001 '**' 0.01 '*' 0.05 '.' 0.1 ' ' 1

##

## exp(coef) exp(-coef) lower .95 upper .95

## SEPSIS 2.716 0.3682 2.628 2.807

##

## Concordance= 0.562 (se = 0.001 )

## Likelihood ratio test= 2827 on 1 df, p=<2e-16

## Wald test = 3553 on 1 df, p=<2e-16

## Score (logrank) test = 3750 on 1 df, p=<2e-16, Robust = 1920 p=<2e-16

##

## (Note: the likelihood ratio and score tests assume independence of

## observations within a cluster, the Wald and robust score tests do not).

crudehr$coef

## SEPSIS

## 0.9992557

exp(cbind( crudehr$coef, confint( crudehr))) ***# Calculation of the Hazard ratio and its confidence intervals***

## 2.5 % 97.5 %

## SEPSIS 2.716259 2.628456 2.806996

Risk functions, separately for SEPSIS = 0 and SEPSIS = 1

stype = survival function estimate the default value in R for survfit is stype = 1, i.e. direct or the Aalen-Johansen approach) ctype = cumulative risk estimate in R, the default value is ctype = 1, which is the Nelson-Aalen formula Meanwhile, ctype = 2, which is the Fleming-Harrington correction for tied events

kmcurve_0 <- survfit(Surv(year, mortality) ~ 1, data = long[long$SEPSIS == 0,], type = "kaplan-meier", conf.type="none")

kmcurve_1 <- survfit(Surv(year, mortality) ~ 1, data = long[long$SEPSIS == 1,], type = "kaplan-meier", conf.type="none")

km <- data.frame(

SEPSIS = c(rep(0, length(kmcurve_0$time) + 1), rep(1, length(kmcurve_1$time) + 1)),

year = c(0, kmcurve_0$time, 0, kmcurve_1$time),

mortality = c(1, kmcurve_0$surv, 1, kmcurve_1$surv),

r = c(0, 1 - kmcurve_0$surv, 0, 1 - kmcurve_1$surv) * 100

)

SGPlot <- ggplot(data = km, aes(x = year, y = r, color = as.factor(SEPSIS))) +

geom_step() +

theme_bw() +

labs(title = " Crude association between SEPSIS and Mortality per year ", color = "SEPSIS")

SGPlot

**
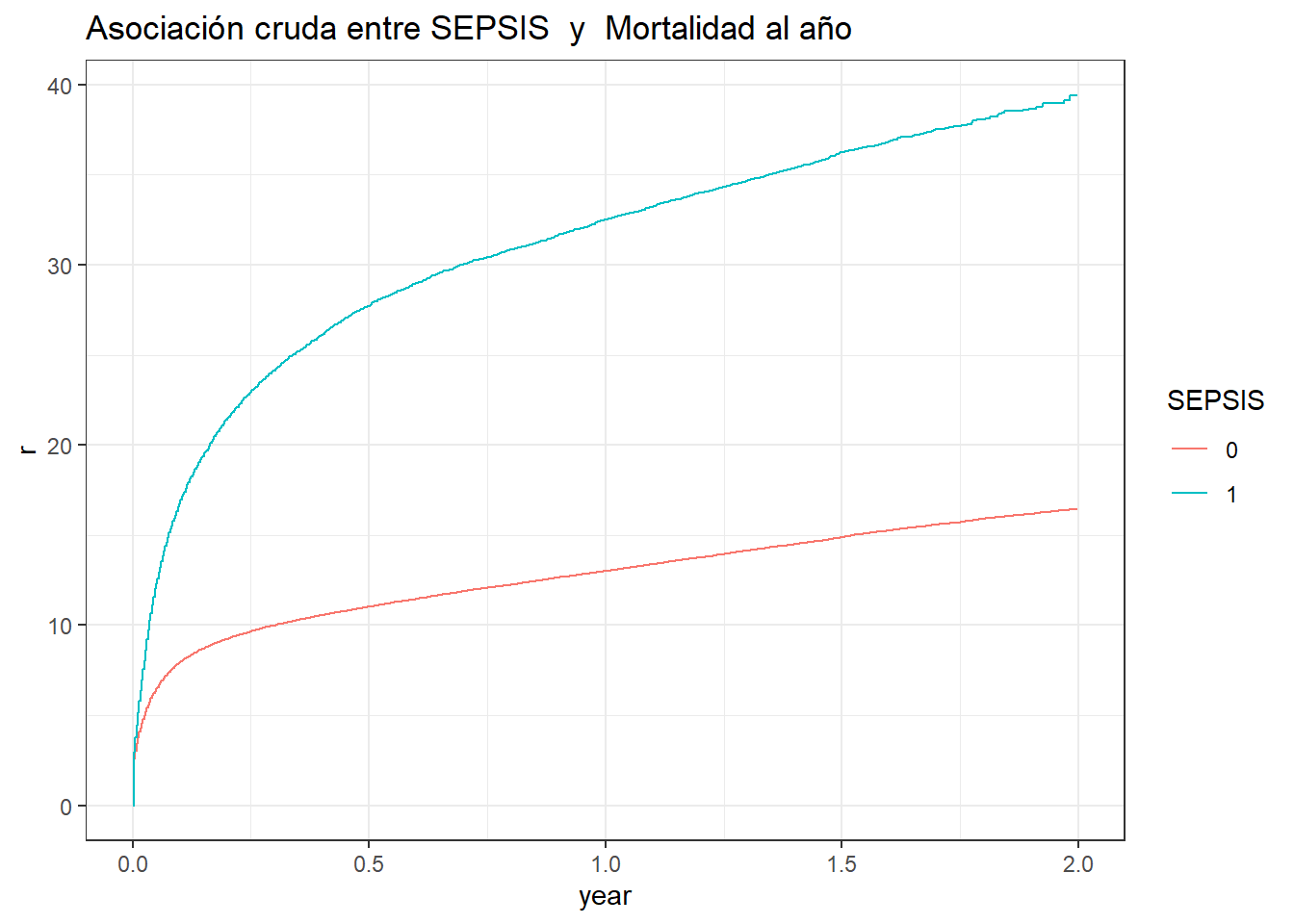
**

ggsave("crude_association.png")

## Saving 7 x 5 in image

Calculate risks, risk difference (RD) and risk ratio (RR)

r <- km %>% group_by(SEPSIS) %>% filter(row_number() == n()) %>% pull()

rd <- r[2] - r[1]

rr <- r[2]/r[1]

result <- c(r, rd, rr)

names(result) <- c("r0", "r1", "rd", "rr")

result

## r0 r1 rd rr

## 16.453344 39.423093 22.969750 2.396054

**Run a Cox proportional hazards model to calculate the covariate-adjusted hazard ratio between SEPSIS and 1-year mortality.**

adjhr <- coxph(Surv(year, mortality) ~ SEPSIS + ACV + ICC + IR + DEME + EPOC + CA + HTA + DM + TC + EVP + IAM + sexo + edad + EH + TM + VIH + PLEJIA , data = long, method = "efron", robust = TRUE, id = personabasicaid)

summary(adjhr)

## Call:

## coxph(formula = Surv(year, mortality) ~ SEPSIS + ACV + ICC +

## IR + DEME + EPOC + CA + HTA + DM + TC + EVP + IAM + sexo +

## edad + EH + TM + VIH + PLEJIA, data = long, robust = TRUE,

## method = "efron", id = personabasicaid)

##

## n= 116329, number of events= 20377

## (123 observations deleted due to missingness)

##

## coef exp(coef) se(coef) robust se z Pr(>|z|)

## SEPSIS 0.5986791 1.8197136 0.0176310 0.0184201 32.501 < 2e-16 ***

## ACV 0.3804304 1.4629141 0.0216720 0.0224697 16.931 < 2e-16 ***

## ICC 0.2461310 1.2790672 0.0346270 0.0347275 7.088 1.37e-12 ***

## IR 1.1551677 3.1745557 0.0203509 0.0215103 53.703 < 2e-16 ***

## DEME 0.0830686 1.0866164 0.0291417 0.0293136 2.834 0.004600 **

## EPOC 0.0646201 1.0667536 0.0167658 0.0170122 3.798 0.000146 ***

## CA 0.7279060 2.0707399 0.0358863 0.0413142 17.619 < 2e-16 ***

## HTA -0.7153640 0.4890141 0.0154543 0.0156269 -45.778 < 2e-16 ***

## DM -0.0401966 0.9606005 0.0163798 0.0164526 -2.443 0.014559 *

## TC 0.1957947 1.2162772 0.0317593 0.0319878 6.121 9.30e-10 ***

## EVP 0.0422833 1.0431900 0.0339759 0.0347759 1.216 0.224030

## IAM -0.1580651 0.8537942 0.0193677 0.0193597 -8.165 3.22e-16 ***

## sexo 0.1071003 1.1130459 0.0142430 0.0143873 7.444 9.76e-14 ***

## edad 0.0486050 1.0498056 0.0005542 0.0006219 78.161 < 2e-16 ***

## EH 0.8000348 2.2256183 0.0470700 0.0530201 15.089 < 2e-16 ***

## TM 0.7014499 2.0166746 0.0277789 0.0292146 24.010 < 2e-16 ***

## VIH 0.4099186 1.5066952 0.0502380 0.0528830 7.751 9.09e-15 ***

## PLEJIA 0.1959268 1.2164379 0.1318282 0.1569635 1.248 0.211946

## ---

## Signif. codes: 0 '***' 0.001 '**' 0.01 '*' 0.05 '.' 0.1 ' ' 1

##

## exp(coef) exp(-coef) lower .95 upper .95

## SEPSIS 1.8197 0.5495 1.7552 1.8866

## ACV 1.4629 0.6836 1.3999 1.5288

## ICC 1.2791 0.7818 1.1949 1.3692

## IR 3.1746 0.3150 3.0435 3.3113

## DEME 1.0866 0.9203 1.0259 1.1509

## EPOC 1.0668 0.9374 1.0318 1.1029

## CA 2.0707 0.4829 1.9097 2.2454

## HTA 0.4890 2.0449 0.4743 0.5042

## DM 0.9606 1.0410 0.9301 0.9921

## TC 1.2163 0.8222 1.1424 1.2950

## EVP 1.0432 0.9586 0.9745 1.1168

## IAM 0.8538 1.1712 0.8220 0.8868

## sexo 1.1130 0.8984 1.0821 1.1449

## edad 1.0498 0.9526 1.0485 1.0511

## EH 2.2256 0.4493 2.0059 2.4693

## TM 2.0167 0.4959 1.9044 2.1355

## VIH 1.5067 0.6637 1.3583 1.6712

## PLEJIA 1.2164 0.8221 0.8943 1.6546

##

## Concordance= 0.754 (se = 0.002 )

## Likelihood ratio test= 17504 on 18 df, p=<2e-16

## Wald test = 17049 on 18 df, p=<2e-16

## Score (logrank) test = 20490 on 18 df, p=<2e-16, Robust = 10711 p=<2e-16

##

## (Note: the likelihood ratio and score tests assume independence of

## observations within a cluster, the Wald and robust score tests do not).

**Calculating the propensity score based on covariates propensity_scores**

propensity_scores <- glm(SEPSIS ~ ACV + ICC + IR + DEME + EPOC + CA + HTA + DM + TC + EVP + IAM + sexo + edad + EH + TM + VIH + PLEJIA , data = long, family = binomial(link = "logit"))

**summary(propensity_scores)**

##

## Call:

## glm(formula = SEPSIS ~ ACV + ICC + IR + DEME + EPOC + CA + HTA +

## DM + TC + EVP + IAM + sexo + edad + EH + TM + VIH + PLEJIA,

## family = binomial(link = "logit"), data = long)

##

## Deviance Residuals:

## Min 1Q Median 3Q Max

## -1.6022 -0.4812 -0.4072 -0.3535 2.5736

##

## Coefficients:

## Estimate Std. Error z value Pr(>|z|)

## (Intercept) -2.8555915 0.0407570 -70.064 < 2e-16 ***

## ACV 0.3350214 0.0335322 9.991 < 2e-16 ***

## ICC 0.2219142 0.0528951 4.195 2.72e-05 ***

## IR 1.0206187 0.0324874 31.416 < 2e-16 ***

## DEME 0.3869646 0.0453166 8.539 < 2e-16 ***

## EPOC 0.5652643 0.0233229 24.236 < 2e-16 ***

## CA 0.7360273 0.0607633 12.113 < 2e-16 ***

## HTA -0.5213037 0.0220179 -23.676 < 2e-16 ***

## DM 0.2855466 0.0229142 12.462 < 2e-16 ***

## TC -0.0149772 0.0460870 -0.325 0.745

## EVP 0.5102803 0.0472157 10.807 < 2e-16 ***

## IAM -0.1530772 0.0281624 -5.436 5.46e-08 ***

## sexo -0.0933645 0.0198931 -4.693 2.69e-06 ***

## edad 0.0108766 0.0006515 16.694 < 2e-16 ***

## EH 0.8393262 0.0759538 11.050 < 2e-16 ***

## TM 0.3979282 0.0459709 8.656 < 2e-16 ***

## VIH 0.6600503 0.0633448 10.420 < 2e-16 ***

## PLEJIA 0.8081071 0.1675198 4.824 1.41e-06 ***

## ---

## Signif. codes: 0 '***' 0.001 '**' 0.01 '*' 0.05 '.' 0.1 ' ' 1

##

## (Dispersion parameter for binomial family taken to be 1)

##

## Null deviance: 77476 on 116328 degrees of freedom

## Residual deviance: 73973 on 116311 degrees of freedom

## (123 observations deleted due to missingness)

## AIC: 74009

##

## Number of Fisher Scoring iterations: 5

long$propensity_score <- predict(propensity_scores, newdata = long, type = "response")

***# Calculate propensity weights***

long$ipw_weight <- ifelse(long$SEPSIS == 1, 1 / long$propensity_score, 1 / (1 -long$propensity_score))

**IPTW-adjusted association between Sepsis and Mortality**

**library**(survival)

***#*** ***Fit the Cox model with IPW***

***#coxph_model <- coxph(Surv(year, mortality) ~ SEPSIS + ACV + ICC + IR + DEME + EPOC + CA + HTA + DM + # TC + EVP + IAM + sexo + edad + EH + TM + VIH + PLEJIA , data = long, weights = long$ipw_weight)***

long$edad_c <- factor(long$edad_c)

coxph_model <- coxph(Surv(year, mortality) ~ SEPSIS , data = long, weights = long$ipw_weight)

*#SEPSIS + edad_c*

summary(coxph_model)

## Call:

## coxph(formula = Surv(year, mortality) ~ SEPSIS, data = long,

## weights = long$ipw_weight)

##

## n= 116329, number of events= 20377

## (123 observations deleted due to missingness)

##

## coef exp(coef) se(coef) robust se z Pr(>|z|)

## SEPSIS 0.742836 2.101888 0.009051 0.019063 38.97 <2e-16 ***

## ---

## Signif. codes: 0 '***' 0.001 '**' 0.01 '*' 0.05 '.' 0.1 ' ' 1

##

## exp(coef) exp(-coef) lower .95 upper .95

## SEPSIS 2.102 0.4758 2.025 2.182

##

## Concordance= 0.589 (se = 0.002 )

## Likelihood ratio test= 7119 on 1 df, p=<2e-16

## Wald test = 1518 on 1 df, p=<2e-16

## Score (logrank) test = 7049 on 1 df, p=<2e-16, Robust = 961.3 p=<2e-16

##

## (Note: the likelihood ratio and score tests assume independence of

## observations within a cluster, the Wald and robust score tests do not).

**Fitted Kaplan Meier curves**

**library**(survival)

***# Converts SEPSIS to a categorical variable***

long$SEPSIS <- factor(long$SEPSIS)

coxph_model <- coxph(Surv(year, mortality) ~ SEPSIS, data = long, weights = long$ipw_weight)

newdata <- data.frame(SEPSIS = levels(long$SEPSIS)) ***# We use SEPSIS levels as input data***

surv_obj <- survfit(coxph_model, newdata = newdata, se.fit = FALSE)

plot(surv_obj, xlab = "Years", ylab = "Probability of survival",

main = "One-year survival stratified by sepsis and weighted by IPW",

col = c("blue", "red"), lty = c(1, 2))

legend("bottomright", legend = c("SEPSIS Negative", "SEPSIS Positive"),

lty = c(1, 2), col = c("blue", "red"))

**
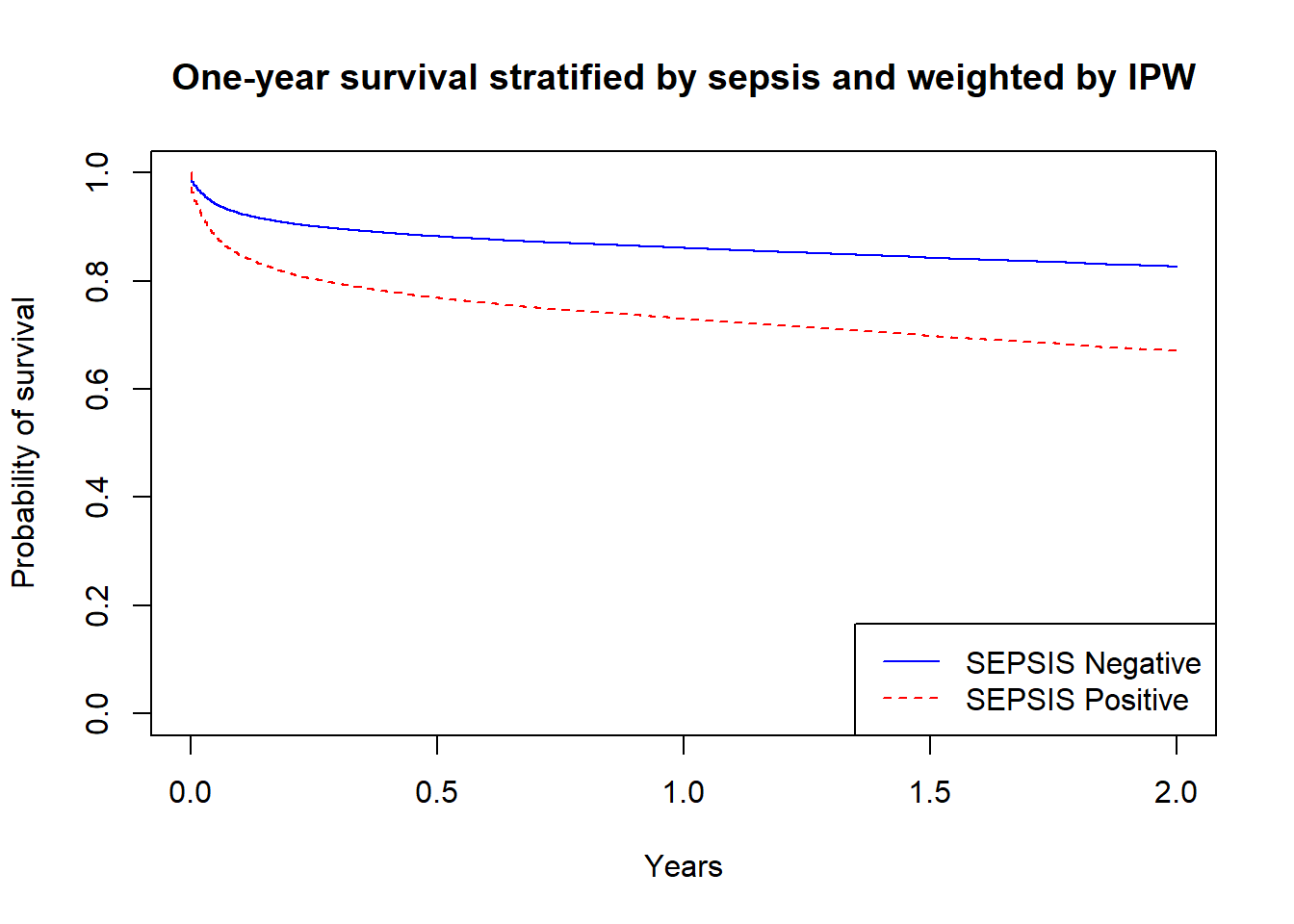
**

**MAP with mortality percentages due to SEPSIS per year**

*#############################################################################################*

**library**(readxl)

## Warning: package 'readxl' was built under R version 4.2.3

**library**(ggplot2)

**library**(sf)

## Warning: package 'sf' was built under R version 4.2.3

## Linking to GEOS 3.9.3, GDAL 3.5.2, PROJ 8.2.1; sf_use_s2() is TRUE

**library**(dplyr)

**library**(rnaturalearth)

## Warning: package 'rnaturalearth' was built under R version 4.2.3

**library**(rnaturalearthdata)

## Warning: package 'rnaturalearthdata' was built under R version 4.2.3

##

## Attaching package: 'rnaturalearthdata'

## The following object is masked from 'package:rnaturalearth':

##

## countries110

setwd("C:/Users/henry/Dropbox/MAPA")

colombia <- ne_states(country = "colombia", returnclass = "sf")

sepsis <- read_excel("C:/Users/henry/Dropbox/MAPA/Datos_sepsis.xlsx",

col_types = c("text", "numeric"))

mapa_colombia <- left_join(colombia, sepsis, by = c("name" = "departamento"))

ggplot() +

geom_sf(data = mapa_colombia, aes(fill = Mortality)) +

scale_fill_gradient(low = "#98F5FF", high = "#0000FF") +

theme_void() +

coord_sf(xlim = c(-82, -66), ylim = c(-4, 13)) +

labs(title = "Mortality at 30 days in patients with sepsis", subtitle = "Colombian Territory - 2019 -"

---

title: "SEPSIS"

author: "Henry Oliveros"

date: "2024-01-18"

output: html_document

---

```{r setup, include=FALSE}

knitr::opts_chunk$set(echo = TRUE)

```

## R Markdown

This is an R Markdown document. Markdown is a simple formatting syntax for authoring HTML, PDF, and MS Word documents. For more details on using R Markdown see <http://rmarkdown.rstudio.com>.

When you click the **Knit** button a document will be generated that includes both content as well as the output of any embedded R code chunks within the document. You can embed an R code chunk like this:

```{r }

{r,echo=FALSE}

library(readxl)

library(ggplot2)

library(sf)

library(rnaturalearth)

library(rnaturalearthdata)

library(dplyr)

```

## Including Plots

```{r}

colombia <- ne_states(country = "colombia", returnclass = "sf")

```

## Including Plots

```{r}

Data_colombia_map <- st_read("ruta/a/tus/datos/departamentos_colombia.shp")

```

## Including Plots

```{r}

colombiaa data <- read_excel("Data_sepsis.xlsx")

col_types = c("text", "numeric")

View(Datos_colombiaa)

```

## Including Plots

```{r}

data_colombia_map <- left_join(colombia, Datos_colombiaa, by = c("name" = "nombre_departamento"))

```

## Including Plots

```{r}

ggplot() +

geom_sf(data = datos_colombia_mapa, aes(fill = `Casos pre`)) +

scale_fill_gradient(low = "#40E0D0", high = "#FF3030") +

theme_void() +

labs(title = " Crude mortality map by departments in Colombia ", subtitle = " departments in Colombia ")

```
